# Peroral endoscopic myotomy in spastic esophageal disorders: Clinical outcomes and optimal approaches

**DOI:** 10.1111/den.15008

**Published:** 2025-03-17

**Authors:** Tetsuya Tatsuta, Haruhiro Inoue, Yuto Shimamura, Miyuki Iwasaki, Kei Ushikubo, Kazuki Yamamoto, Yohei Nishikawa, Hidenori Tanaka, Ippei Tanaka, Satoshi Abiko, Mayo Tanabe, Kazuya Sumi, Manabu Onimaru, Boldbaatar Gantuya, Hirotake Sakuraba, Shinsaku Fukuda

**Affiliations:** ^1^ Digestive Diseases Center Showa University Koto‐Toyosu Hospital Tokyo Japan; ^2^ Department of Gastroenterology, Hematology and Clinical Pathology Hirosaki University Graduate School of Medicine Aomori Japan

**Keywords:** gastroesophageal reflux disease, lower esophageal sphincter preservation, peroral endoscopic myotomy, spastic esophageal disorder

## Abstract

**Objectives:**

The efficacy and safety of peroral endoscopic myotomy (POEM) for spastic esophageal disorders (SED), including type III achalasia, distal esophageal spasm (DES), and jackhammer esophagus (JE), remain uncertain due to its rarity. This study aimed to evaluate the clinical outcomes and optimal strategies for managing SED.

**Methods:**

A retrospective analysis was conducted on patients who underwent POEM for SED between March 2014 and December 2023. Myotomy was tailored to target spastic segments in all cases. For type III achalasia, the myotomy extended into the gastric cardia, while for DES and JE, the procedure either preserved the lower esophageal sphincter (LES) or included a gastric myotomy. Outcomes assessed included procedural details, technical and clinical success, adverse events, and the incidence of gastroesophageal reflux disease (GERD) post‐POEM. Clinical success was defined as an Eckardt score of ≤3.

**Results:**

Among 2938 POEM procedures, 106 (3.6%) were for SED. The cohort included 58 patients (54.8%) with type III achalasia, 24 (22.6%) with DES, and 24 (22.6%) with JE. The technical success rate was 100%, with clinical success rates of 98.1% at 2–3 months and 92.6% at 1 year. Erosive esophagitis occurred in 27.7% at 2–3 months and 16.1% at 1 year. LES‐preserving POEM for DES and JE showed comparable efficacy to POEM with gastric myotomy, with a trend toward reduced GERD incidence.

**Conclusion:**

Peroral endoscopic myotomy is an effective treatment for all types of SED. LES‐preserving POEM is a viable strategy for treating DES and JE, offering comparable efficacy, while potentially minimizing GERD risk.

## INTRODUCTION

Peroral endoscopic myotomy (POEM) has become the standard treatment for achalasia.^1^ However, its efficacy in managing spastic esophageal disorders (SED), including distal esophageal spasm (DES) and hypercontractile esophagus, such as jackhammer esophagus (JE), remains unclear due to the rarity of these conditions.

Current treatment options for SED include pharmacological therapies, such as nitrates, calcium channel blockers, anticholinergics, and antidepressants.^2^, ^3^ Additionally, pneumatic dilation and botulinum toxin injections have been applied,^4^, ^5^, ^6^ although these approaches often provide limited and transient relief, especially for chest pain, which can severely impair patients' quality of life. Surgical myotomy has shown satisfactory outcomes in 80% of patients,^7^ but its invasive nature and technical challenges of performing an esophageal myotomy have limited its widespread adoption.

While some studies have explored the treatment of SED subtypes, few have specifically investigated the role of POEM in these disorders, and current guidelines reflect this uncertainty. The European Society of Gastrointestinal Endoscopy (ESGE) advises caution when performing POEM for SED other than achalasia,^8^ whereas the Japan Gastroenterological Endoscopy Society (JGES) provides weak recommendations for its use in nonachalasia motility disorders.^9^ Despite limited data, POEM is endorsed as the preferred treatment for type III achalasia by both the American Gastroenterological Association's Clinical Practice Update and an international expert consensus statement.^10^ A meta‐analysis evaluating POEM for SED, including type III achalasia, DES, and JE, confirmed its efficacy and safety as a therapeutic option.^11^ Compared to surgical myotomy, POEM offers a minimally invasive approach with the advantage of tailored myotomy length. High‐resolution manometry (HRM)‐tailored POEM for type III achalasia has demonstrated superior outcomes compared to nontailored procedures.^12^ Despite these advancements, questions remain regarding the optimal strategy for SED, particularly concerning myotomy length and the preservation of the lower esophageal sphincter (LES).^13^

This study aimed to evaluate the clinical outcomes of POEM in SED and determine the optimal treatment strategy, focusing on the potential benefits of LES preservation.

## METHODS

### Patient selection

This retrospective analysis utilized prospectively collected data from two tertiary referral centers: Showa University Koto Toyosu Hospital (Tokyo, Japan) and Hirosaki University (Aomori, Japan), covering the period from March 2014 to December 2023. Patients who underwent POEM for type III achalasia and nonachalasia esophageal motility disorders, including DES and JE, diagnosed via HRM were included. Preprocedure evaluations comprised barium esophagogram, esophagogastroduodenoscopy (EGD), and computed tomography. Patients without a definite diagnosis based on these evaluations were excluded. However, those with a history of endoscopic dilation, botulinum toxin injections, or laparoscopic surgical myotomy were included. This study was conducted in compliance with the Declaration of Helsinki and was approved by the Research Ethics Committees of Showa University (approval number 2024‐132‐B) and Hirosaki University Hospital (approval number 2024‐044).

### High‐resolution manometry

All HRM examinations were performed using the Starlet system (Star Medical, Tokyo, Japan). Diagnosis of type III achalasia, DES, and hypercontractile esophagus including JE was based on the Chicago Classification version 4.0 criteria.^14^ Type III achalasia was diagnosed by an elevated median integrated relaxation pressure (IRP) (>26 mmHg)^15^ without normal peristalsis, accompanied by premature (spastic) contractions with a distal contractile integral (DCI) exceeding 1000 mmHg·s·cm in **≥**20% of swallows.^16^ Premature contractions were identified by a distal contractile latency <4.5 s, measured as the interval from the upper esophageal sphincter relaxation to the contractile deceleration point.^16^ JE was characterized by a normal mean IRP (≤26 mmHg) and hypercontractility, with a DCI **≥**10,000 mmHg·s·cm in ≥20% of swallows.^16^ DES was defined by a normal mean IRP and premature contractions in ≥20% of swallows during a standard 10‐water swallow test.^16^

### Procedure of POEM

POEM was performed under general anesthesia with endotracheal intubation, with patients positioned supine. A single‐channel therapeutic endoscope (GIF‐Q260J or GIF‐290T; Olympus, Tokyo, Japan) with CO_2_ insufflation was used throughout the procedure. Submucosal tunneling and myotomy were performed using a TriangleTip knife (KD‐640L or KD‐645L; Olympus) mainly in Spray Coagulation mode. Hemostasis and coagulation of larger vessels were achieved with coagulation forceps (Coagrasper FD‐410LR; Olympus) in soft coagulation mode. The mucosal entry site was closed with endoclips (EZ Clip HX‐110LR or QuickClip Pro; Olympus). The distal end of the submucosal tunnel was confirmed using the double‐scope method in most cases.^17^ Myotomy length was determined based on HRM findings, targeting the spastic segment on the esophageal side. For type III achalasia, the myotomy extended into the gastric side. In DES and JE cases, myotomy was performed either with preservation of the LES or with a gastric myotomy.

### Evaluation of treatment effect

Technical success was defined as the successful completion of the myotomy. Treatment efficacy was evaluated 2–3 months post‐POEM through assessments including Eckardt score evaluation, HRM, EGD, and barium esophagogram. For patients who were available for follow‐up at 1 year, symptom improvement was reassessed using the Eckardt score and EGD. The use of proton pump inhibitor (PPI) therapy during follow‐up was determined based on the patients' symptoms. Notably, for EGD assessments, patients were not required to discontinue PPI therapy prior to the procedure. Clinical success was defined as symptom improvement, evidenced by an Eckardt score of ≤3 at both the 2–3 month and 1‐year follow‐ups after POEM.^18^ For DES and JE cases, the relationship between the length of the gastric‐side myotomy and treatment efficacy was further analyzed. The safety of the POEM procedure was assessed by categorizing adverse events using the Clavien–Dindo classification.^19^ Pneumoperitoneum findings that did not impact perioperative outcomes were not considered adverse events. The diagnosis of gastroesophageal reflux disease (GERD) was based on the presence of typical GERD symptoms or endoscopic findings of erosive esophagitis graded above Los Angeles Classification grade B during follow‐up EGD.^20^ The incidence of GERD was assessed for type III achalasia, DES, and JE, with a specific focus on the association between gastric myotomy length and GERD development in DES and JE cases.

### Statistical analysis

Continuous variables are presented as median (interquartile range; IQR), while categorical variables are expressed as frequency counts and percentages. Differences between groups were assessed using Fisher's exact test for categorical data. The Mann–Whitney *U*‐test was applied to compare independent continuous data medians, and the Wilcoxon signed‐rank test was used to compare paired medians of continuous data. Two‐sided *P*‐values <0.05 were considered statistically significant. All statistical analyses were performed using EZR.^21^

## RESULTS

### Patient demographics

A total of 2938 POEM procedures were performed during the study period for achalasia and nonachalasia esophageal motility disorders. Among 118 cases presenting with esophageal motility disorders characterized by spasm and hypercontractility, 12 cases without a definite diagnosis for either subtype were excluded. Consequently, 106 patients (median age 68 years [IQR 56–75 years], 70 men and 36 women) were included in the analysis. The manometric subtypes were as follows: 58 patients (54.81%) had type III achalasia, 24 patients (22.6%) had DES, and 24 patients (22.6%) had JE.

### Clinical outcomes

POEM was technically successful in all patients (106/106). The median procedural time was 97 min (IQR 77–120). Anterior myotomy was performed in 42 patients (39.6%), while posterior myotomy was performed in 64 patients (60.4%). The median length of esophageal and gastric myotomies were 12 cm (IQR 9–16 cm) and 2 cm (IQR 1–2 cm), respectively (Table 1). The double‐scope method was performed in 75 cases (70.7%). The median follow‐up duration was 12 months (IQR 3–25). Follow‐up EGD was conducted in 94 cases (88.7%) at 2–3 months post‐POEM, and in 56 cases (52.8%) at 1 year. Significant improvement in the Eckardt score was observed 2–3 months post‐POEM, decreasing from 5.0 (IQR 3–8) to 0.5 (IQR 0–2) (*P* < 0.05). The overall clinical success rate 2–3 months after POEM was 98.1% (104/106), with subtype‐specific success rates of 98.3% (57/58) for type III achalasia, 100% (24/24) for DES, and 95.8% (23/24) for JE (Table 1). The overall clinical success rate at 1 year was 92.6% (50/54). At 2–3 months after POEM, 26/94 patients (27.7%) exhibited erosive esophagitis (Los Angeles Classification grade B and above) on endoscopy, while 26/92 (28.3%) reported typical GERD symptoms. PPI use was reported in 21.5% (20/93) of patients. At 1 year, 9/56 (16.1%) had erosive esophagitis, and 12/55 (21.8%) reported typical GERD symptoms, and 15/54 (27.8%) were taking PPIs. Adverse events, classified according to the Clavien–Dindo classification as grade ≤ IIIa, occurred in three patients (2.8%), including pneumothorax, perforation, and submucosal hematoma, all managed conservatively. One JE patient developed severe symptomatic GERD requiring surgical fundoplication. Peroral endoscopic fundoplication^22^ was performed in DES patients with GERD symptoms after POEM. A redo POEM was performed in three patients (one each with type III achalasia, JE, and DES) due to poor postoperative symptom improvement.

**Table 1:** Clinical outcomes of peroral endoscopic myotomy (POEM)

	Total (*n* = 106)	Type III achalasia (*n* = 58)	JE (*n* = 24)	DES (*n* = 24)
Operation time (min), median (IQR)	97 (77–120)	95 (71–120)	109 (90–135.5)	97 (76.5–107.5)
Length of myotomy				
Esophageal side (cm), median (IQR)	12 (9–16)	12 (9–15.8)	12 (10.3–16.5)	12 (9.5–16.5)
Gastric side (cm), median (IQR)	3.0 (2.0–3.0)	2.0 (2.0–2.0)	1.5 (0–2.0)	1.0 (0–2.0)
Eckardt score				
Before POEM, median (IQR)	5.0 (3–8)	6 (4–8)	4 (3–5)	5 (3–8)
2–3 months after POEM, median (IQR)	0.5 (0–2)	0.5 (0–2)	0 (0–1)	1 (0–1.3)
1 year after POEM, median (IQR)	1 (0–2)	2 (1–2.3)	0 (0–1)	0 (0–1.5)
Clinical success				
2–3 months after POEM, *n* (%)	104 (98.1%)	57 (98.3%)	23 (95.8%)	24 (100%)
1 year after POEM, *n* (%)	50/54 (92.6%)	29/32 (90.6%)	10/11 (90.9%)	11/11 (100%)
Adverse events, *n* (%)	3 (2.8%)	2 (3.4%)	0 (0%)	1 (4.2%)
IRP				
Before POEM, median (IQR)	19.9 (13.0–31.0)	25.4 (18–35.3)	14.4 (8.4–18.6)	17.3 (13.9–27.4)
2–3 months After POEM, median (IQR)	10.3 (7.1–14.2)	10.3 (6.8–13.5)	9.3 (7.4–14.2)	10.4 (8.1–16.7)
GERD after POEM (2–3 months post‐POEM)				
GERD LA grade classification, *n*	N: 47, A: 21 B: 19, C: 5, D: 2	N: 26, A: 13 B: 11, C: 2, D: 2	N: 12, A: 4 B: 3, C: 1	N: 9, A: 4 B: 5, C: 2
≥Grade B, *n* (%)	26/94 (27.7%)	15/54 (27.8%)	4/20 (20%)	7/20 (35%)
GERD symptoms, *n* (%)	26/92 (28.3%)	17/52 (32.7%)	5/20 (25%)	4/20 (20%)
PPI intake, *n* (%)	20/93 (21.5%)	9/52 (17.3%)	5/20 (25%)	6/21 (28.6%)
GERD after POEM (1 year post‐POEM)				
GERD LA grade classification, *n*	N: 34, A: 13 B: 8, C: 1	N: 18, A: 10 B: 4	N: 8, A: 1 B: 2	N: 8, A: 2 B: 2, C: 1
≥Grade B	9/56 (16.1%)	4/32 (12.5%)	2/11 (18.2%)	3/13 (23.1%)
GERD symptoms, *n* (%)	12/55 (21.8%)	9/32 (28.1%)	1/11 (9.1%)	2/12 (16.7%)
PPI intake, *n* (%)	15/54 (27.8%)	9/31 (29.0%)	3/11 (27.3%)	3/12 (25%)

DES, distal esophageal spasm; GERD, gastroesophageal reflux disease; IQR, interquartile range; IRP, integrated relaxation pressure; JE, jackhammer esophagus; LA grade, Los Angeles Classification; PPI, proton pump inhibitor.

### Comparative outcomes by subtype and myotomy length

When comparing clinical outcomes between type III achalasia and the combined DES + JE group, no significant differences were observed in procedure time, clinical success rates, or adverse event rates. The gastric myotomy length was significantly shorter in the DES + JE group compared to the type III achalasia group (DES + JE: 1.0 cm [0–2.0] vs. type III: 2.0 cm [2.0–2.0], *P* < 0.05; Table 2). For DES and JE cases, LES‐preserving POEM demonstrated similar efficacy to standard POEM with gastric myotomy. Patients were categorized into three groups based on the myotomy length from the squamocolumnar junction, confirmed using the double‐scope method: LES preservation (*n* = 7), 0 cm and 1 cm (*n* = 17), and 2 cm and 3 cm (*n* = 21). IRP significantly decreased post myotomy in gastric myotomy cases, while no significant decrease was observed in LES‐preserving cases (Table 3). Median Eckardt scores showed significant symptom improvement across all groups (Table 4). Treatment success rates 2–3 months after POEM were 7/7 (100%) for LES preservation, 17/17 (100%) for the 0 cm and 1 cm group, and 20/21 (95.2%) for the 2 cm and 3 cm group, indicating no difference in efficacy despite shorter myotomy lengths. At 2–3 months post‐POEM, the incidence of erosive esophagitis was 1/6 (16.7%) in the LES‐preservation group, 5/14 (35.7%) in the 0 cm and 1 cm group, and 5/17 (29.4%) in the 2 cm and 3 cm groups, with a nonsignificant trend toward a lower incidence in the LES‐preserving group. This showed similar trend in 1‐year follow‐up (Table 4).

**Table 2:** Comparison between type III achalasia and distal esophageal spasm (DES) + jackhammer esophagus (JE)

	Type III achalasia (*n* = 58)	DES + JE (*n* = 48)	*P*‐value
Operation times (min), median (IQR)	95 (71–120)	100.5 (80.0–129.3)	0.38
Myotomy length			
Esophageal side (cm), median (IQR)	12 (9–15.8)	12.0 (10.0–17.0)	0.493
Gastric side (cm), median (IQR)	2.0 (2.0–2.0)	1.0 (0–2.0)	<0.05
Eckardt score			
Before POEM, median (IQR)	6 (4–8)	4.0 (3.0–7.0)	0.116
2–3 months After POEM, median (IQR)	0.5 (0–2)	0.5 (0–1.0)	0.485
Clinical success			
Treatment success, *n* (%)	57 (98.3%)	47 (97.9%)	1
Adverse events, *n* (%)	2 (3.4%)	1 (2.1%)	1
IRP			
Before POEM, median (IQR)	25.4 (18–35.3)	16.3 (11.7–20.3)	<0.05
2–3 months After POEM, median (IQR)	10.3 (6.8–13.5)	10.2 (7.6–15.0)	0.211
GERD after POEM (2–3 months post‐POEM)			
GERD LA grade classification, *n*	N: 26, A: 13 B: 11, C: 2, D: 2	N: 19, A: 7 B: 8, C: 3	
GERD symptoms, *n* (%)	17/52 (32.7%)	9/40 (22.5%)	0.353
≥Grade B, *n* (%)	15/54 (27.8%)	11 /40 (27.5%)	1

*P*‐value was calculated by Mann–Whitney *U*‐test for continuous data and Fisher's exact test for categorical data.

GERD, gastroesophageal reflux disease; IQR, interquartile range; IRP, integrated relaxation pressure; LA grade, Los Angeles Classification; POEM, peroral endoscopic myotomy.

**Table 3:** Integrated relaxation pressure (IRP) changes based on the length of myotomy

	IRP, median (IQR)		*P‐*value
	Before POEM	2–3 months after POEM	
LES preservation (*n* = 6)	18.5 (12.3–24.1)	15.8 (10.3–25.5)	0.844
Gastric myotomy 0 cm and 1 cm (*n* = 12)	16.6 (9.3–17.8)	11.5 (8.6–14.5)	<0.05
Gastric myotomy 2 cm and 3 cm (*n* = 14)	14.5 (12.7–26.2)	8.65 (6.8–12.4)	<0.05

*P‐*value was calculated by Wilcoxon signed‐rank test.

IQR, interquartile range; LES, lower esophageal sphincter; POEM, peroral endoscopic myotomy.

**Table 4:** Clinical outcomes based on the length of myotomy

	LES preservation (*n* = 7)	Gastric myotomy 0 and 1 cm (*n* = 17)	Gastric myotomy 2 and 3 cm (*n* = 21)	*P*‐value
Chicago classification	DES: 4 JE: 3	DES: 9 JE: 8	DES: 10 JE: 11	
Eckardt score				
Before POEM, median (IQR)	5.0 (3.5–7.5)	3.0 (3.0–5.0)	4.5 (4.0–5.8)	0.378
2–3 months after POEM, median (IQR)	0 (0–1.0)	1.0 (0–1.0)	0 (0–2.0)	0.773
1 year after POEM, median (IQR)	0 (0–0)	1.5 (0.3–2)	0 (0–1)	0.369
Clinical success				
2–3 months after POEM, *n* (%)	7/7 (100%)	17/17 (100%)	20 /21 (95.2%)	1
1 year after POEM, *n* (%)	4/5 (80%)	6/6 (100%)	10/10 (100%)	0.714
GERD after POEM				
GERD ≥ LA classification B				
2–3 month after POEM, *n* (%)	1/6 (16.7%)	5/14 (35.7%)	5/17 (29.4%)	0.891
1 year after POEM, *n* (%)	1/5 (20%)	2/6 (33.3%)	2/10 (20%)	0.804
PPI intake				
2–3 months after POEM, *n* (%)	2/7 (28.6%)	6/14 (42.9%)	3/17 (17.6%)	0.465
1 year after POEM, *n* (%)	1/5 (20%)	1/6 (16.7%)	3/10 (30%)	0.849

*P‐*value was calculated by Fisher's exact test.

DES, distal esophageal spasm; GERD, gastroesophageal reflux disease; IQR, interquartile range; JE, jackhammer esophagus; LA, Los Angeles; LES, lower esophageal sphincter; POEM, peroral endoscopic myotomy; PPI, proton pump inhibitor.

## DISCUSSION

In this study, POEM demonstrated favorable short‐term outcomes in 98.1% of patients with type III achalasia, DES, and JE, with no severe adverse events reported. Among these patients, 26/94 (27.7%) exhibited endoscopic evidence of erosive esophagitis, aligning with the known risk of GERD following POEM. Our findings revealed no significant difference in treatment effectiveness between LES‐preserving POEM and POEM with gastric myotomy for DES and JE. However, there was a nonsignificant trend suggesting a lower incidence of GERD with the LES‐preserving approach.

Previous studies have reported a POEM success rate of 90.6% (29/32) in type III achalasia patients, with mean pretreatment and posttreatment Eckardt scores of 7.2 and 1.4, respectively (*P* < 0.001).^23^ Another study found clinical response rates of 96.3% in type III achalasia, 100% in DES, and 70% in JE across 73 patients (54 with type III achalasia, 9 with DES, and 10 with JE).^24^ Our results are consistent with or exceed these reported outcomes. As discussed, the key advantage of POEM over surgical myotomy is its minimally invasive nature and the ability to tailor the myotomy length based on the most proximal spastic segment identified by HRM. This individualized approach likely contributes to the excellent outcomes observed and emphasizes the importance of tailored treatment for these subtypes.

Differentiating type III achalasia from other spastic disorders is crucial because, in type III achalasia, both the esophageal body and impaired LES relaxation are affected, necessitating an adequate gastric myotomy during POEM. However, the optimal length of gastric myotomy for DES and JE remains unclear, and few studies have addressed whether the LES should be preserved during POEM for these conditions. In one case of JE treated with POEM (myotomy length 12 cm) in 2015, without confirmation of the submucosal tunnel orientation via the double‐scope method, the patient experienced significant symptom improvement but developed severe, PPI‐refractory reflux esophagitis, requiring Toupet fundoplication after antireflux mucosectomy failed to resolve the symptoms. As a result, we currently favor LES‐preserving or short gastric myotomy in DES and JE patients.

**Figure 1:**
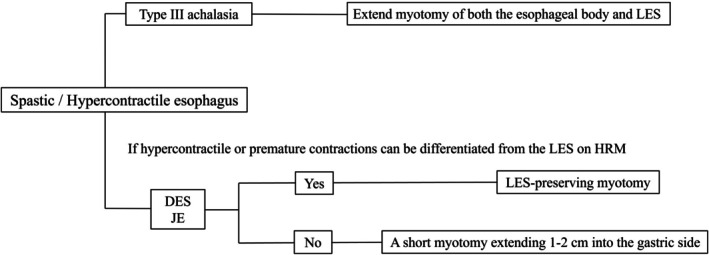
Treatment algorithm for spastic esophageal disorders. For type III achalasia, extended myotomy of both the esophageal body and lower esophageal sphincter (LES) is necessary. In cases of distal esophageal spasm (DES) and jackhammer esophagus (JE), if hypercontractile or premature contractions can be differentiated from the LES on high‐resolution myotomy (HRM), peroral endoscopic myotomy (POEM) with LES preservation is recommended. If symptoms persist after LES‐preserving POEM, a redo POEM may be considered. When differentiation is not possible, a short myotomy extending 1–2 cm into the gastric side is considered sufficient.

**Figure 2:**
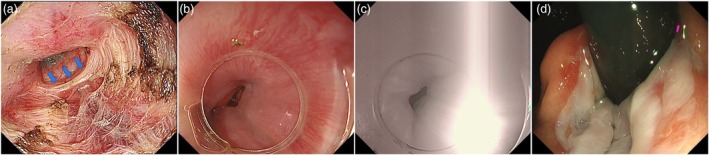
Peroral endoscopic myotomy (POEM) with lower esophageal sphincter (LES) preservation. (a) For distal esophageal spasm (DES) and jackhammer esophagus (JE) myotomy, we terminate the incision when the inner circular muscle becomes thin (blue arrow). (b, c) Double‐scope method around the lower esophageal sphincter (LES). Transillumination was confirmed at the level where the palisade vessels are visible, ensuring that the incision did not extend to the LES. (d) The scope‐holding sign in the retroflex view was used for additional verification.

Figure 1 illustrates our current treatment algorithm for SED. For DES and JE patients where hypercontractile or premature contractions can be distinguished from the LES on HRM, LES‐preserving POEM can be considered. To achieve LES preservation, the myotomy was terminated when the inner circular muscle became sufficiently thin, avoiding extension into the gastric side (Fig. 2a). The distal extent of the submucosal tunnel was confirmed using the double‐scope method (Fig. 2b,c), ensuring precise and adequate myotomy without excessive extension. The scope‐holding sign, observing LES function under excessive insufflation in the retroflex view, was also used for verification (Fig. 2d).^25^, ^26^ LES‐preserving myotomy was defined as one limited to the esophageal side, confirmed by scope measurement, the double‐scope method, and a positive scope‐holding sign. When differentiation is not possible, a short myotomy extending 0 cm and 1 cm into the gastric side is considered sufficient, as confirmed by the double‐scope method. This controlled approach is preferred to avoid severe GERD, which may be refractory to medication and other endoscopic therapies. Our study demonstrated comparable treatment efficacy among the three groups: LES preservation, 0 cm and 1 cm gastric myotomy, and 2 cm and 3 cm gastric myotomy. The occurrence of blown‐out myotomy, a focal increase in the lumen diameter around LES after myotomy due to high pressure with poor bolus clearance, has been previously reported in LES‐preserving POEM, but was not observed in our study.^27^ These results suggest that LES‐preserving POEM or short gastric myotomy is sufficient for treating DES and JE as long as LES is confirmed with the aforementioned methods. In addition, we have not encountered any case of severe GERD since adopting this strategy. If symptoms persist after LES‐preserving POEM, a redo POEM may be a better option than managing severe GERD.

Although we did not observe a statistically significant reduction in GERD prevalence with shorter gastric myotomy, there was a trend toward lower GERD incidence post‐POEM. GERD grade B and above was observed 2–3 months after POEM in 27.7% of the entire cohort (26/94), with 27.8% (15/54) in the type III achalasia subgroup and 27.5% (11/40) in the DES + JE group. A multicenter study reported a post‐POEM GERD incidence of ~30%,^28^ making our observed GERD incidence comparatively low. Recent studies on POEM with LES preservation have reported a 93.8% success rate (15/16) in patients with DES and JE, with GERD (LA grade A/B) observed in 12.5% (2/16).^13^ In our study, the clinical success rate 2–3 months after POEM was 100%, with a 16.7% GERD (grade B and above), indicating the acceptability of this approach. Although some follow‐up data were missing, the 1‐year outcomes demonstrated comparable efficacy.

Our study had several limitations. First, being a retrospective analysis, it carried inherent risks of selection bias and missing data. Notably, patients who did not return for follow‐up, particularly for the 1‐year follow‐up assessments, were excluded, which may have limited the comprehensiveness of our findings. Additionally, the number of LES‐preserving POEM cases was relatively small compared to POEM with gastric myotomy, which may have affected the robustness of our conclusions. Further evaluation with a larger, dedicated cohort is necessary to thoroughly assess the safety and efficacy of LES‐preserving POEM. Furthermore, the POEM procedure evolved throughout the study period as operators gained experience and refined techniques, and the length of myotomy was decided at the discretion of the operator, leading to variability in treatment strategies that could have influenced the consistency of the observed outcomes.

In conclusion, POEM is an effective and safe treatment for patients with SED. LES‐preserving POEM is a viable strategy for treating DES and JE, offering comparable treatment efficacy with potentially reduced GERD risk.

## CONFLICT OF INTEREST

Author H.I. serves as an advisor to Olympus Corporation and Top Corporation and has received educational grants from Olympus Corporation and Takeda Pharmaceutical Co. Author H.S. serves as an Associate Editor for *Digestive Endoscopy*. The other authors declare no conflict of interest for this article.

## FUNDING INFORMATION

None.

## ETHICS STATEMENT

Approval of the research protocol by an Institutional Reviewer Board: The study was approved by the Institutional Review Board of Showa University (approval number 2024‐132‐B) and Hirosaki University Hospital (approval number 2024–044).

Informed Consent: N/A.

Registry and the Registration No. of the study/trial: N/A.

Animal Studies: N/A.
